# A New Machine Learning Algorithm for Numerical Prediction of Near-Earth Environment Sensors along the Inland of East Antarctica

**DOI:** 10.3390/s21030755

**Published:** 2021-01-23

**Authors:** Yuchen Wang, Yinke Dou, Wangxiao Yang, Jingxue Guo, Xiaomin Chang, Minghu Ding, Xueyuan Tang

**Affiliations:** 1 College of Electrical and Power Engineering, Taiyuan University of Technology, Taiyuan 030024, China; wangyuchen0204@link.tyut.edu.cn (Y.W.); yangwangxiao0038@link.tyut.edu.cn (W.Y.); 2 SOA Key Laboratory for Polar Science, Polar Research Institute of China, Shanghai 200136, China; guojingxue@pric.org.cn (J.G.); tangxueyuan@pric.org.cn (X.T.); 3 College of Water Resources Science and Engineering, Taiyuan University of Technology, Taiyuan 030024, China; changxiaomin@tyut.edu.cn; 4 State Key Laboratory of Severe Weather and Institute of Tibetan Plateau & Polar Meteorology, Chinese Academy of Meteorological Sciences, Beijing 100081, China; dingmh@cma.gov.cn

**Keywords:** neural network, East Antarctica, multi-sensor, LSTM

## Abstract

Accurate short-term small-area meteorological forecasts are essential to ensure the safety of operations and equipment operations in the Antarctic interior. This study proposes a deep learning-based multi-input neural network model to address this problem. The newly proposed model is predicted by combining a stacked autoencoder and a long- and short-term memory network. The self-stacking autoencoder maximises the features and removes redundancy from the target weather station’s sensor data and extracts temporal features from the sensor data using a long- and short-term memory network. The proposed new model evaluates the prediction performance and generalisation capability at four observation sites at different East Antarctic latitudes (including the Antarctic maximum and the coastal region). The performance of five deep learning networks is compared through five evaluation metrics, and the optimal form of input combination is discussed. The results show that the prediction capability of the model outperforms the other models. It provides a new method for short-term meteorological prediction in a small inland Antarctic region.

## 1. Introduction

Research and logistical activities in and around Antarctica are heavily dependent on environmental forecasting systems’ reliable forecasts. The establishment of an integrated multi-sensor environmental prediction system is an urgent task when conducting scientific research activities and station area construction, especially in areas where local weather forecasts are lacking. Prediction of wind speed and temperature has been an issue of particular interest. However, due to the unique geography of East Antarctica, real-time forecasting is difficult in small areas. The existing meteorological stations and sensor data processing methods are mostly numerical models. The Australian Bureau of Meteorology uses the Community Climate System Simulation Global (ACCESS-G) Characteristic Numerical Atmospheric Weather Prediction (NWP) suite for Antarctic weather prediction. However, forecasts in the extreme high latitudes of the southeast have been shown to have severe degradation of model performance in the high southern latitudes in the validation work of Schroeter et al. 2019 [1]. However, when calculating large models, the hardware’s performance requirements are relatively high due to a large amount of data and high time complexity [2]. Numerical calculations may be unreliable in the field or in emergencies where rapid prediction results are required. In addition to this, environmental prediction methods for East Antarctica’s interior are less common in various studies, mainly because of the high altitude and harsh environment of the region, which makes sensor data collection difficult. This study aims to obtain a machine learning method for analyzing and predicting data from the surface atmospheric weather sensor array in the eastern part of Antarctica.

To obtain new data-driven methods and essential technical methods of data prediction, researchers in related fields have introduced machine learning methods in the process of surface environment prediction and data analysis. This method has been applied in many cases and achieved scientific goals. With machine learning development, image recognition techniques were applied to atmospheric observations [3]. However, under Antarctica’s harsh climatic conditions and communication difficulties, sensors with lower power consumption become the preferred solution using less data transmission capacity. In addition to this, the lower air visibility index directly affects optical imaging. As a result, the device described in the article cannot work.

Yeh et al. 2019 combines sensor fusion with neural network prediction to improve urban wind turbine maintenance efficiency in various environments [4]. Similar work has been done at the Spanish National Meteorological Service (AEMET) to establish a network of meteorological stations on eight islands in the Canary Islands. Using data from these weather stations, Javier et al. 2019 propose an innovative machine learning added value based on a data stream mining paradigm. It was used to predict the wind speed in the region [5]. In the wind signature recognition study, Leopoldo et al. 2017 applied genetic algorithms to in-flight airspeed sensor data to make predictions [6]. Furthermore, in wind prediction in ships, Mei et al. 2020 used pattern search algorithms to predict wind and current rate [7]. In emerging work on spatial feature extraction for multidimensional, multi-location time series, Bilgera et al. 2018 used the CNN-LSTM algorithm to estimate gas source locations [8]. The prophecy of wind speed plays a vital role in predicting the Antarctic region’s meteorological environment. High winds in the middle and high altitude areas of East Antarctica sometimes cause equipment damage.

In addition to predicting wind speed, the prediction of temperature is an important task. Surface temperature measurements in the East Antarctic are mainly based on temperature sensors in weather stations. Still, because the main research stations in the region are near the coastline, there is little access to meteorological data in the East Antarctic interior. Because of the East Antarctic’s specific geographic area, most of the temperature prediction work and sensor data processing methods remain in the West Antarctic. Many of the work using machine learning in West Antarctica is mostly single-input neural networks, and there will be problems such as gradient disappearance during practical training. This makes it difficult to extract data features in the learning process. In surface climate prediction in the West Antarctic, David et al. 2005 use feed-forward neural networks to reconstruct ice cores for annual mean climate conditions based on surface weather station and shallow snow core data [9]. This method was the first approach to combining neural networks to predict surface temperatures. To alleviate the shortage of Antarctic surface meteorological data and make better use of this valuable data resource, David et al. 2004 use artificial neural network-based techniques to extend and fill data gaps in selected records from automated weather stations [10]. It can be seen that machine learning is one of the practical and popular methods for supplementing data and predicting data in the Antarctic.

Hochreiter and Schmidhuber proposed a temporally recurrent neural network in 1997, and their proposed network is known as the Long Short Term Memory (LSTM) network. The LSTM does not require exceptional complexity to debug hyperparameters, and it can choose to remember or forget long-term information through a forgetting gate [11]. Many researchers have used LSTM as a practical algorithm for solving vanishing gradients in electricity pricing, stock prices, robot control and disease prediction [12]. For time series prediction and analysis, this algorithm outperforms other traditional machine learning algorithms.

In deep learning methods, stacked autoencoders (SAEs), typically learn high-level features incrementally from low-level features by minimising each input layer [13]. The technique is widely used in the field of soft perception. There are also corresponding variants that have been applied in the process, such as the deep-stacked isomorphic autoencoder (SIAE), which was used to improve soft measurement methods for prediction by Yan et al. 2020 [14]. In addition to this, a deep correlation learning method (DRRL) based on superimposed self-encoders was proposed by Yuan et al. 2020 to address feature representation techniques for complex structured data 1 [15]. Combining various applications, the advantage of SAE stands out in its ability to capture hidden features. This is a clear advantage in cases where the data volume is small, and the parts are not prominent. The acquisition of components is essential because of the scarcity of data in the Antarctic. The introduction of this method has aided in the learning and capture of features.

In the second part, the article presents measurement data from four stations with different meteorological conditions from the East Antarctic coast to the highest point of Dome A and the types of sensor data. The third part focuses on the LSTM and Bi LSTM neural networks, and the joint training prediction method of neural network SAE with neural network LSTM. In the fourth section of the article, the authors compare the prediction performance of five neural networks, reflecting the results in terms of error statistics; in the fifth section of the article, the authors discuss the prediction performance and time spent by each model. The final section of the article summarises the study’s innovations and main work and provides an outlook for future work.

## 2. Study Areas and Materials

### 2.1. Areas of Study

**Figure 1:**
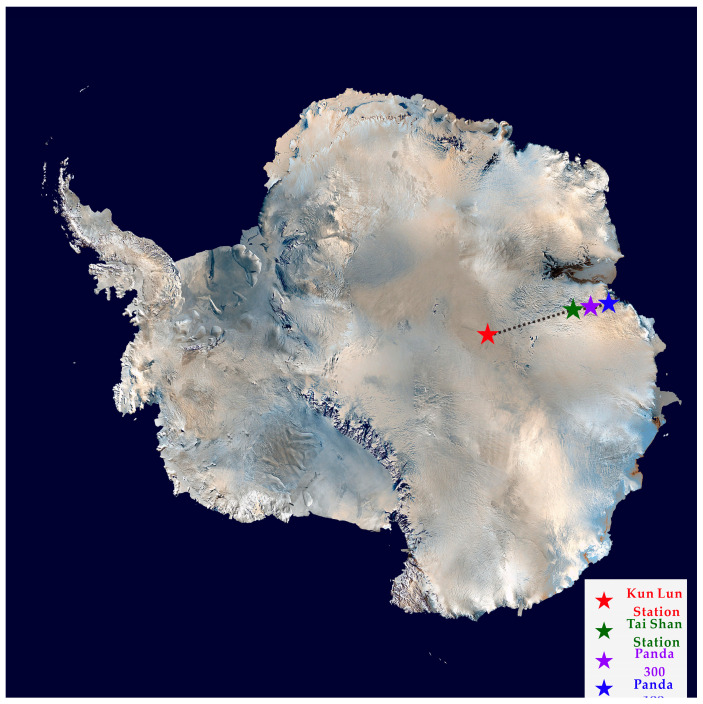
Position distribution of the four stations.

Research in the East Antarctic has focused on the edges of ice shelves, areas of visible surface rock and sizes of ice domes, such as the highest point in Antarctica, Dome A, and areas such as the Princess Elizabeth Land. Four unmanned station observation sites were selected at different altitudes and under other environmental conditions to test the algorithm’s stability. The meteorological stations were laid out in positions and the Chinese Antarctic Scientific Expedition (PANDA route). These include sites with geographical characteristics. As shown in Figure 1, the four star-shaped markers on the Antarctic continent represent the geographical locations of the four groups of sensor sites in this study, including the 100 km from the coast area, the 300 km from the coast area, the Princess Elizabeth Land area and the Dome A Antarctic Highest Point area. Data provided by the Chinese Academy of Meteorological Sciences. The detailed layout of the four monitoring stations is shown in Table 1.

### 2.2. Input Parameters and Correlation Analysis

**Figure 2:**
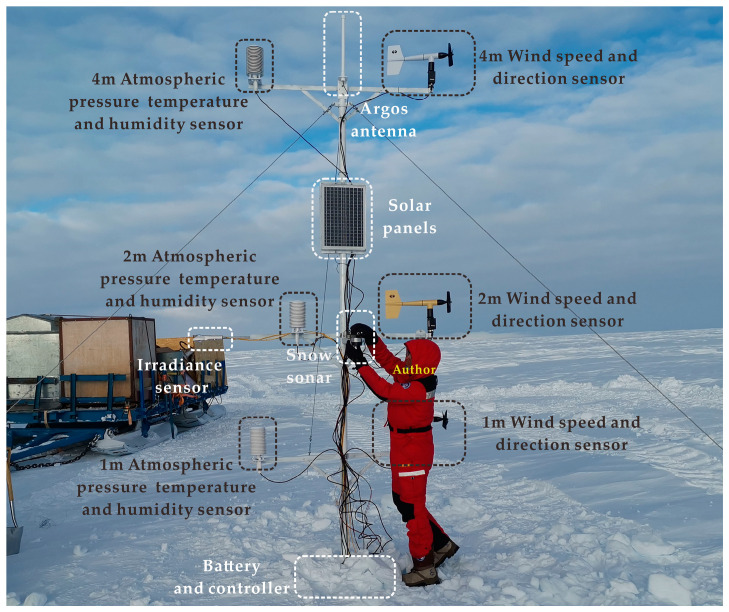
Photo of the author’s fieldwork during the installation of the Panda 300 weather station on the way to an inland scientific expedition on 13 December 2019.

Meteorological observations are needed to analyse the course of wind and temperature changes in an area. The panda 300 weather station at 300 km on the inner route was placed on 13 December 2019, as shown in Figure 2. The observable factors include air temperature and humidity at 1 m, air temperature and humidity at 2 m and air temperature and humidity at 4 m. wind speed and direction at 1 m, wind speed and direction at 2 m and wind speed and direction at 4 m. Light irradiance and snow surface albedo, snow depth and snow quality. As the sensor at 1 m on the surface will encounter snow burial, ground disturbance and other factors during operation, the correlation analysis will not be carried out due to the loss of data in multiple sections of the sensor at 1 m, and the data on light irradiance and snow accumulation is not publicly available. Since changes in temperature and wind speed are relatively unlike snow depth, etc., which take a long time to accumulate, the temperature and wind speed and direction are simply predicted. Changes in each parameter’s values up to 1 h, each parameter up to 2 h and each parameter over a more extended period are not considered. The correlation coefficient $R_{x,y}$ between the input parameters can be expressed as:

(1) $$R_{x,y}=\frac{\mathrm{cov}(x,y)}{\sigma_{x}\sigma_{y}}=\frac{\frac{1}{n}\sum_{n}^{i=1}(x_{i}-\overset{ˉ}{x})(y_{i}-\overset{ˉ}{y})}{\sqrt{\frac{1}{n}\overset{i=1}{\underset{n}{\sum }}{\left(x_{i}\;-\;\overset{ˉ}{x}\right)}^{2}}\sqrt{\frac{1}{n}\overset{i=1}{\underset{n}{\sum }}{\left(y_{i}-\overset{ˉ}{y}\right)}^{2}}}$$

In Equation (1), x and y represent two different environmental variables, cov(x,y) represents the covariance between them.σ represents the root mean square.

**Figure 3:**
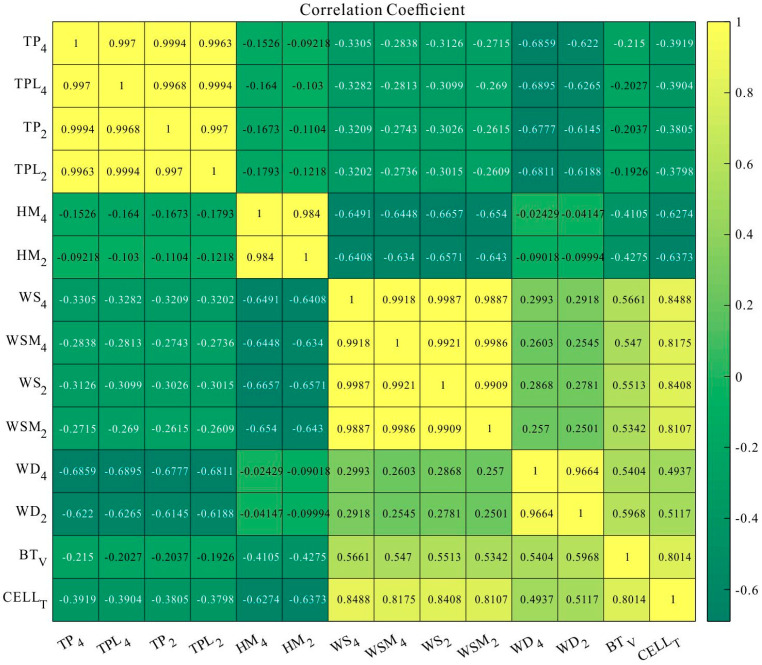
Correlation diagram of the nine input parameters of the deployed weather station (weather station panda 300 for example).

The correlation diagram for the primary nine parameters is shown in Figure 3. TP_4_ and TP_2_ represent the mean values of the temperatures at 4 m and 2 m per hour, respectively (the mean values are calculated by averaging the values collected every ten minutes for one hour, the same applies to the means below), TPL_4_ and TPL_2_ represent the lowest temperatures collected per hour at 4 m and 2 m, respectively. HM_4_ and HM_2_ represent the mean value of humidity per hour at 4 m and 2 m, respectively, WS_4_ and WS_2_ represent the mean value of wind speed per hour at 4 m and 2 m, respectively, WD_4_ and WD_2_ represent the mean value of wind direction per hour at 4 m and 2 m, respectively, and WSM_4_ and WSM_2_ represent the maximum wind speed collected per hour at 4 m and 2 m, respectively. BT_V_ and CELL_T_ represent the battery voltage as well as the controller temperature under the snow. As the Antarctic climate is a non-linear process, parameters are selectively screened when selecting input features. Correlation colour charts of the relevant parameters are, therefore, analysed. The closer the correlation coefficient in the colour chart is to 1, the more similar the features’ trend is. Such a combination of input variables is often chosen for prediction during multivariate input training of neural networks.

### 2.3. Normalisation and Regularisation

The dataset used in this study precedes the model training. Some work needs to be done to prepare the data. As most of the sensors used to collect the data were installed in December 2019, the job of supplementing a large amount of missing data can be omitted, and only the denoising of data and the supplement of individual data points are required. The data were collected over four months and, except for a few missing data, the total number of data samples per weather station was around 2900. The selection of the input dataset for the neural network model was based on two considerations: firstly, the human peer window for the Antarctic Inland Route was considered to be in December and April; secondly, the requirement for model training efficiency was that the model training time had to be within 20 min. With these two considerations in mind, the dataset was chosen for January–April. One of the prediction phases was the error analysis of the April data. In the input data of the neural network model 2175 samples (75%) was used as a training set for the experiment and 725 samples (25%) as a test set (the model does not adjust the parameters for the results to achieve the effect of changing the hyperparameters, and the test set does not show over- and under-fitting of the prediction results, so a separate validation set is not necessary). The purpose of this experiment is to compare the prediction performance of the newly proposed machine learning algorithm with other machine learning algorithms on the same data set. It is not an optimization problem for a particular algorithm. The separate partitioning of the validation set has been omitted to save time. The data’s normalisation is the scaling of the data to fall into a small, specific interval. This is transformed into pure, dimensionless values so that indicators of different units or magnitudes can be compared and weighed. A single sample’s characteristics are subtracted from the average of all training samples (same features) and divided by all training samples’ variance. The features of individual segments are deducted from the average of all training samples (same characteristics) and divided by all training samples’ conflict. Thus, for each fragment, all data are clustered around 0 with a variance of 1. This is calculated as follows:

(2) $$X(normal)=\frac{x-\mu }{\sigma }$$

In Equation (2), where *x* is the sample eigenvalue, *μ* is the sample mean, *σ* is the standard deviation of the sample data, and *X* is the normalised eigenvalue [16].

One of the goals of neural network prediction is to normalise parameters while minimising prediction error. To minimise the error is to get the predicted value closer to the training data, and to normalise the parameters is to prevent over-fitting. The leading cause of overfitting is too many parameters. The error in the test set of the predictions is much larger than the error in the training set. The “simplicity” of the model is ensured to minimise training errors and give good generalisation performance of the resulting parameters. The fitting process usually tends to make the weights as small as possible and construct a model with small values for all parameters. This is because it is generally accepted that models with small parameter values are simpler, adapted to different datasets, and avoid overfitting. In addition to using the L_1_ regularisation method, the L_2_ regularisation method is used to prevent overfitting. In this paper, the L_2_ regularisation method is used, with the standard term Equation (3), limiting the training model’s complexity.

(3) $$J=J_{0}+\alpha \sum w^{2}$$

In Equation (3), *J*_0_ is the original loss function, the latter term is the L2 regularisation term, and *α* is the regularisation factor.

**Figure 4:**
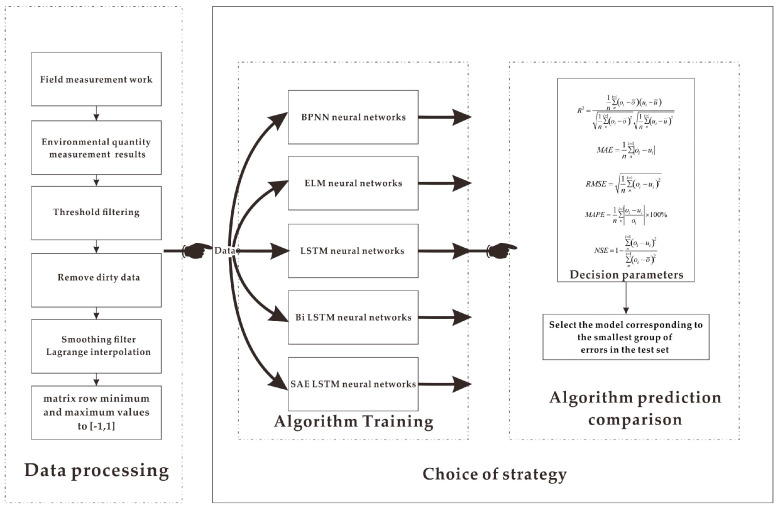
Data cleaning, standardization before data input into the network, and machine learning training and preliminary results comparison flowchart.

The data acquisition process of the equipment in the field is as follows. Each sensor is collected at 10-min intervals, and the values contained each time are stored in Campbell’s data logger. The embedded program compares and averages the data over one hour. In the individual data returns, the data are all real data. Inevitably, there will be missing data and incorrect data collection. The work to be done before input to the neural network for training is data cleaning. This work is also known as pre-processing, and the process involved is shown in Figure 4. The program smoothes out the noisy data from the original data and filters out the irrelevant data. The method used in this study is interpolation to deal with missing values. An interpolation function is created that replaces the unknown values with known points around the missing points. The process is as follows.

## 3. Methodology

In developing and predicting data, RNN has been proposed since the 1980s and has gradually gained widespread application. However, several subsequent problems made RNNs unsuitable for most conditions and situations. Later, LSTM neural networks were developed to solve the problem of gradient disappearance. At the same time, several variants of LSTM have grown accordingly. For example, Bi LSTM, SAE LSTM and some other methods have been proposed to enhance feature learning efficiency.

### 3.1. Long Short-Term Memory Prediction Model

**Figure 5:**
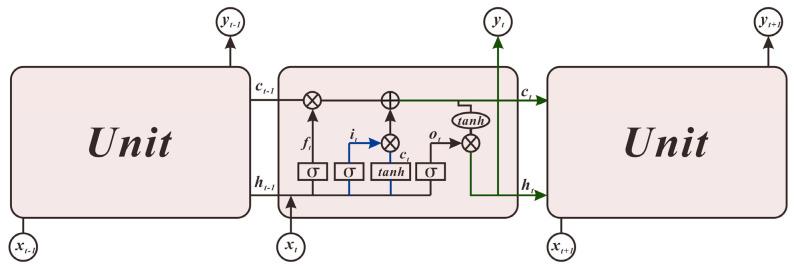
The internal structure of the LSTM neural network unit.

**Figure 6:**
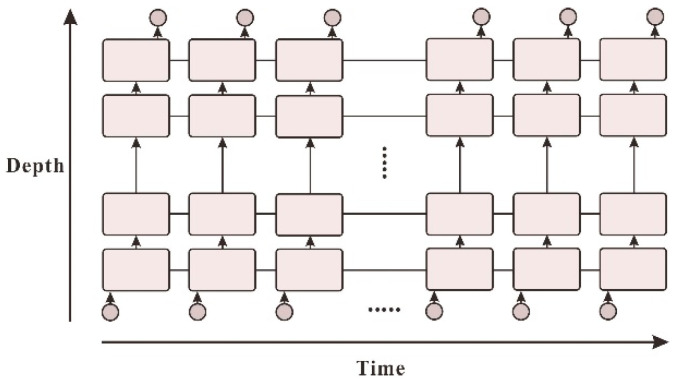
Structural diagram of Long Short-Term Memory Network [18].

A new neural network called LSTM, has been proposed [17], known as Long Short-Term Memory (LSTM) and is used to predict and analyse time-series data. The weather station data obtained in this study include temperature, humidity, barometric pressure and wind speed, all of which are time series, and the three core operator structures contained in LSTM determine how long and short-term memory can be achieved based on RNN The form of the algorithm is shown in Figure 5. The overall operation of the neural network is shown in Figure 6.

The forgotten gate is the process of choosing to forget and is represented as follows:

(4) $$f_{t}=\sigma \left(W_{f}*\left[h_{t-1},x_{t}\right]+b_{f}\right)$$

The input gate selects the information to be entered into the unit. The input gates are represented as follows:

(5) $$\left\{ \begin{array}{l} i_{t}=\sigma \left(W_{i}*\left[h_{t-1},x_{t}\right]+b_{i}\right)\\ c_{t}=f_{t}*c_{t-1}+i_{t}*\tan h\left(W_{c}*\left[h_{t-1},x_{t}\right]+b_{c}\right) \end{array}\right.$$

The output gates are represented as follows:

(6) $$\left\{ \begin{array}{l} o_{t}=\sigma \left(W_{0}*\left[h_{t-1},x_{t}\right]+b_{o}\right)\\ h_{t}=o_{t}*\tan h\left(c_{t}\right) \end{array}\right.$$

In Equation (4), $f_{t}$ is the output of the forgotten gate, and the $W_{f}$ matrix determines the vector of input weights. $b_{f}$ is the bias vector of the forgotten gate; $h_{t-1}$ is the hidden layer state at the last moment; the current input is $x_{i}$, and σ is the activation function where $W_{f}*\left[h_{t-1},x_{i}\right]$ is calculated as shown in Equation (7).

(7) $$W_{f}*\left[h_{t-1},x_{i}\right]=\left[W_{f}\right]*\left[\begin{array}{c} h_{t-1}\\ x_{t} \end{array}\right]=\left[\begin{array}{cc} W_{fh} & W_{fx} \end{array}\right]*\left[\begin{array}{c} h_{t-1}\\ x_{t} \end{array}\right]=W_{fh}h_{t-1}+W_{fx}x_{t}$$

The output of the previous moment in Equation (5) is $h_{t-1}$. It is the value of the current input, $c_{t}$ is the activation state in the current cell, the cell state at the last moment of the previous phase is $c_{t-1}$, $W_{i}$ matrix is the weight in the input gate; $W_{c}$ matrix is the weight in the forgotten gate. $b_{i}$ is the input gate’s bias vector; $b_{c}$ is the bias vector of the forgotten gate.

In Equation (6), $O_{t}$ is the vector in the output gate, $h_{t}$ is the result of the output gate, and the $W_{0}$ matrix is the weight in the output gate. $b_{o}$ is the offset vector in the output gate.

### 3.2. Stacken Autoencoder

The autoencoder is an unsupervised neural network model that learns the input data’s implicit features, called coding, and reconstructs the original input data with the new features known, called decoding. As well as performing feature downscaling, the new features learned by the autoencoder can be fed into a supervised learning model so that the autoencoder can act as a feature extractor. There are many different autoencoders, including stacked autoencoders, under-complete autoencoders, regular autoencoders and so on. AE (autoencoder) [19] has only one hidden layer. More specifically, the AE input and output layers are equal.

**Figure 7:**
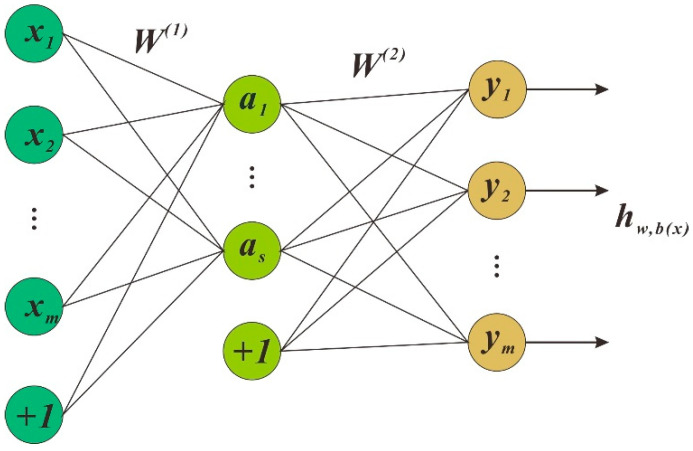
The architecture of an AE.

As shown in Figure 7, the first layer is the input layer. The middle layer is the hidden layer. The last layer is the output layer The AE network can be non-linearly transformed from one layer to the next utilizing an activation function. The encoder converts the input into a more abstract feature vector, and the decoder reconstructs the information from the feature vector. There are two processes in the AE execution, encoding and decoding. The Equations (8) and (9) used in these two processes are shown below:

(8) $$h=f_{1}\left(x_{1}\right)=s_{f1}\left(W_{1}x_{1}+b_{1}\right)$$

(9) $$x_{2}=f_{2}(h)=s_{f2}\left(W_{2}h+b_{2}\right)$$

where $x_{1}={\left[x_{11},x_{12},\cdots ,x_{1dl}\right]}^{T}\in R^{1\mathrm{dl}}$ $h={\left[h_{1},h_{2},\cdots ,h_{dh}\right]}^{T}\in R^{dh}$ is the connection vector between $x_{1}$ and $x_{2}$; $x_{2}={\left[x_{21},x_{22},\cdots ,x_{2dl}\right]}^{T}\in R^{2\mathrm{dr}}$; $b_{1}\in R^{1dl}$ and $b_{2}\in R^{2dr}$ are the bias vector; the sigmoid function is used as $sf_{1}$ and $sf_{2}$.

Obtain the parameters in AE by executing the following Equation (10).

(10) $$J\left(w_{1},w_{2},b_{1},b_{2}\right)=\sum_{i=1}^{N}\left\|x_{2}-x_{1}\right\|/2N=\sum_{i=1}^{N}\left\|g_{\theta }\left(x_{2}\right)-x_{1}\right\|/2N$$

**Figure 8:**
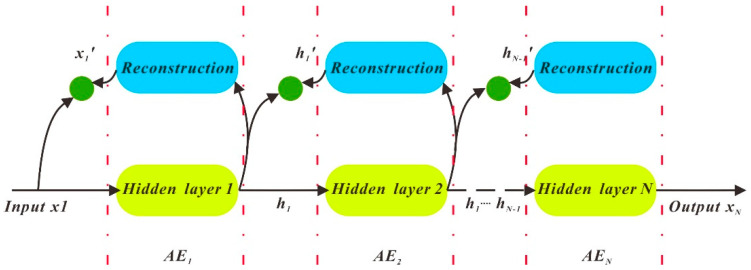
SAE structure diagram.

SAE is a superposition of several AEs. After the first AE has been executed, the subsequent AEs are executed in sequence up to the Nth, with SAE’s output result. The structure of SAE is displayed in Figure 8.

### 3.3. Combination Algorithm for SAE and LSTM with Multiple Inputs

To capture more LSTM neural network features, this study combines SAE and LSTM, the aim of which is to avoid gradient explosion while allowing features in the time series to be learned more fully. The integration of algorithms is divided into four steps:

1. The time series is partitioned into a training set, a test set and a prediction set, and the partitioned series are normalised.
2. They are setting the neural network parameters. For the entire experiment, all neural networks run in the same hardware and software environment.
3. The SAE neural network is first trained to learn the multi-input network features in the hidden layer thoroughly. The results of the SAE features are exported to the LSTM neural network. In phase 3, the SAE performed unsupervised pre-training and supervised fine-tuning. This means that only the SAE model is trained and Equations (8)–(10) are cycled once to obtain one AE output. To get the hidden layer output of the second AE, the production of the previous AE becomes the input of the following AE. The number of training sessions is set to obtain the learned feature matrix of the last AE. The fine-tuning of the entire network utilizing the constraint Equation (11) is performed at this point by using backpropagation, the aim being to obtain improved weights.

(11) $$E_{o}=\overset{N_{l}}{\underset{i=1}{\sum }}\left\|A_{i}-F_{i}\right\|/2N_{l}$$

*N_l_* represents the number of samples; *A_t_* is the actual values and *F_t_* is the prediction value [20].

In the fourth step, the main focus is on the execution of the LSTM algorithm. Since the previous SAE training results are available, the input obtained at SAE combined with multiple input variables is used as input to the LSTM neural network. The output of the products is obtained by getting *f_t_* by Equations (4) and (7), respectively, and by bringing *f_t_* into Equation (5), where it can be obtained. *c_t_* is obtained by substituting *i_t_* and *f_t_* into Equation (5). *o_t_* is obtained by Equation (6).

### 3.4. Bi-Directional Long Short-Term Memory

LSTM is a method for predicting the next moment based on information from past times. In some cases, however, the time series of the current time is not only related to the last moment but may also be related to future moments. In general, the information in an LSTM network is unidirectional, with all learning features coming from the past. LSTM can take into account both history and future data information. LSTM connects two networks on the same principle. The forward LSTM has access to information about the input sequence’s past data, and the backward LSTM has access to information about the future data of the input sequence. This can be expressed in the following formula [21]. Can be represented by the following formula:

(12) $$\left\{ \begin{array}{l} \vec{h_{tf}}=\vec{LSTM}\left(W_{1}h_{t-1},W_{2}x_{t},c_{t-1}\right),t\in [1,T]\\ \overset{\leftarrow }{h_{tb}}=\overset{\leftarrow }{LSTM}\left(W_{3}h_{t+1},W_{4}x_{t},c_{t+1}\right),t\in [T,1]\\ H_{t}=\left[\vec{h_{tf}},\overset{\leftarrow }{h_{tb}}\right] \end{array}\right.$$

The hidden layer state *h_t_* of Bi LSTM at time *t* consists of forwarding *h_tf_* and backward *h_tb_*. The four matrices *W*_1_, *W*_2_, *W*_3_ and *W*_4_ are weighting factor matrices for the hidden layer and the inputs, respectively; *x_t_* is the input sequence at time *t* and *h_t_* is the output of the hidden layer at time *t*. The hidden layer state of Bi LSTM at time *t* consists of forwarding *h_tf_* and backward *h_tb_*.

**Figure 9:**
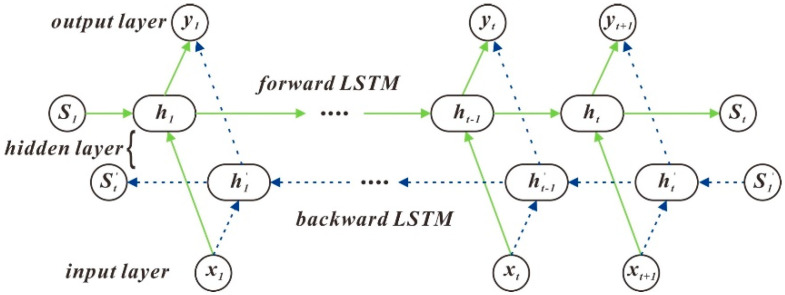
Bi LSTM algorithm structure diagram.

The algorithm structure is shown in Figure 9:

## 4. Simulation & Results

### 4.1. Experimental Design and Parameterisation

The experiment’s preparatory stages are the setting up of the environment and the setting up of the parameters. The location up of the environment consists of a hardware environment and a software environment. The set of parameters includes the neighbourhood of five neural network parameters. The experiments were conducted to compare different prediction targets and additional prediction steps. Table 2 shows the parameters for the computer hardware environment as well as the software environment. To ensure that the experiments are fair, the hardware and software environments used are identical. The quantities were constant in each experiment.

In order to reflect the prediction effect, the correlation coefficient (*R*^2^) [22], root mean square error (*RMSE*) [23], mean absolute error (*MAE*) [24], mean percentage absolute error (*MAPE*) [25] and Nash–Sutcliffe efficiency coefficient (*NSE*) [26] are evaluated. These are given in Equations (13)–(17), respectively:

(13) $$R^{2}=\frac{\frac{1}{n}\sum_{n}^{i=1}(o_{i}-\overset{ˉ}{o})(u_{i}-\overset{ˉ}{u})}{\sqrt{\frac{1}{n}\sum_{n}^{i=1}{\left(o_{i}-\overset{ˉ}{o}\right)}^{2}}\sqrt{\frac{1}{n}\sum_{n}^{i=1}{\left(u_{i}-\overset{ˉ}{u}\right)}^{2}}}$$

(14) $$RMSE=\sqrt{\frac{1}{n}\sum_{n}^{i=1}{\left(o_{i}-u_{i}\right)}^{2}}$$

(15) $$MAE=\frac{1}{n}\sum_{n}^{i=1}\left|o_{i}-u_{i}\right|$$

(16) $$MAPE=\frac{1}{n}\sum_{n}^{i=1}|\frac{o_{i}-u_{i}}{o_{i}}|\times 100\%$$

(17) $$NSE=1-\frac{\sum_{n}^{i=1}{\left(o_{i}-u_{i}\right)}^{2}}{\sum_{n}^{i=1}{\left(o_{i}-\overset{ˉ}{o}\right)}^{2}}$$

where $u_{i}$ is the predicted value, $\bar{u}$ is the average of the expected value, $o_{i}$ is the observed value, and $\bar{o}$ is the average of the experimental values.

The *R*^2^ value is between 0 and 1, where 0 means the model does not explain any variation and one means it explains the observed variation perfectly; *MAE* is mean absolute error, a commonly used error statistic; *RMSE* (root-mean-square error), also known as the standard error, is the squared deviation of the observed value from the actual value. The square root of the ratio of the number of observations. The root mean squared error is a measure of the deviation between an observed value and the real value. The standard error is susceptible to very large or very small errors in a set of measurements and is, therefore, a good indicator of the measure’s precision. The standard error can be used as a criterion for assessing the accuracy of this measurement process; the mean absolute percentage error (*MAPE*), similar to *MAE*, can also be used to measure how well a model predicts results; the *NSE* takes a value from negative infinity to 1, with an *NSE* close to 1 indicating that the model is of good quality and that the model is of good quality. High reliability: an *NSE* close to 0 means that the simulation results are immediate to the mean level of the observations, i.e., the overall results are credible, but the process simulation errors are enormous; an *NSE* less than 0 means that the model is not reasonable.

Table 3 lists the parameter settings of five neural network algorithms. Among them, MEp stands for Max Epochs, GTd stands for Gradient Threshold, H1 stands for Hidden Layer 1, H2 stands for Hidden Layer 2, AF stands for Activation Function, ILR stands for Initial Learn Rate, LRDP stands for Learn Rate Drop Period, LRDF stands for Learn Rate Drop Factor, and Vb stands for Verbose, ETF stands for Encoder Transfer Function, DTF stands for Decoder Transfer-Function, L2R stands for L2Regularization, L2WR stands for L2 Weight Regularization, and SP stands for Sparsity Proportion.

The research results aim to provide a camp meteorological indicator for inland convoys. This is the future wind speed and direction. As the Chinese inland convoy’s daily mileage is 75 km–120 km, the stopping point is the area around each weather station. The driving time during the day is about 7 h. Therefore, it is essential to obtain wind speed and temperature data for the camping site after 7 h to ensure the safety and schedule of the convoy. Once stationed, it is also essential to have hourly weather data for the future. This means organising the operation of the personnel after camping and the loading and unloading of goods. The experiment was, therefore, designed by dividing the whole investigation into 16 group projects. There are four experiments for each station. These are the 4 m temperature prediction for the next hour, the temperature prediction for the next 8 h, the 4 m wind speed prediction for the next hour and the 4 m wind speed prediction for the next 8 h.

### 4.2. Experiment One—Multiparameter Predictions for the Next Hour

**Figure 10:**
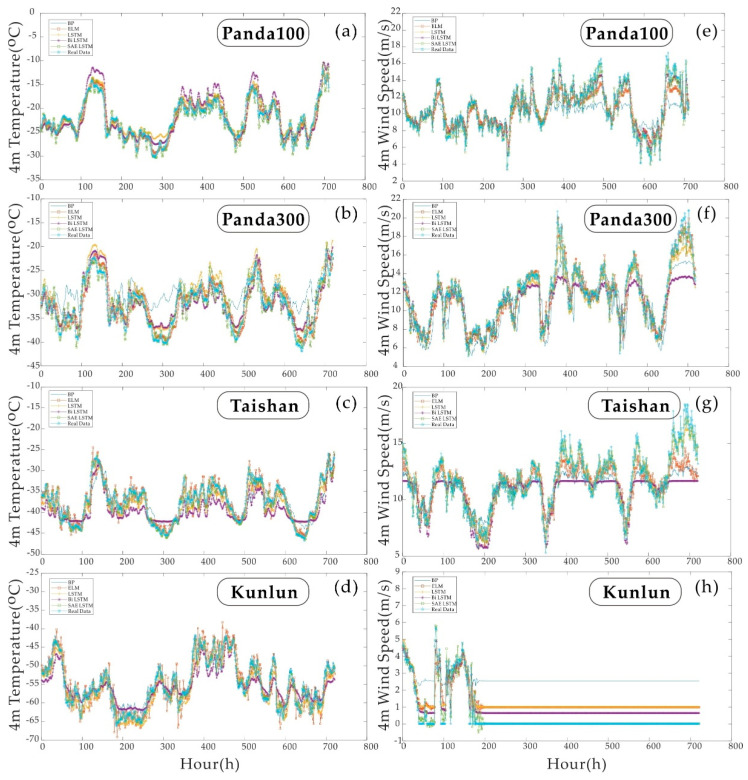
Results of the single-step predictions: (**a**–**d**) are the results of the 4 m temperature single-step predictions for the four sites. (**e**–**h**) are the results of a single-step prediction of the 4 m wind speed at the four areas.

Table 4 and Table 5 show the results of the single-step forecast for each of the four sites. This section contains two sets of experiments. The two sets of experiments are the 4 m temperature one-step prediction comparison at the four areas and the 4 m wind speed one-step prediction comparison at the four sites. The results are compared based on five evaluation indicators: *R*^2^, *RMSE*, *MAE*, *MAPE* and *NSE*. Figure 10 shows a comparison between the new model and four commonly used neural network models on an hourly basis, where (a)–(d) in Figure 10 are the single-step temperature predictions for the four sites. Real Data represents actual environmental parameters. The rest of the lines are the predictions of the deep learning prediction algorithm. The worst predicted model at Pand100 is the Bi LSTM model, which is generally higher than the actual value. Pand300 reflects the situation where the BP neural network indicates much smaller peaks than the actual results. Taishan station reflects the status where the LSTM and Bi LSTM models are compared. Taishan Station reported that the prediction results of LSTM and Bi LSTM models are around 300 h. The prediction results between 600–700 h are not precise, and the magnitude of change is smaller than the real situation. The Kunlun station reflects that the ELM model can have a much higher peak than the actual values during the forecasting process.

Figure 10e–h, are single-step wind speed predictions for the four stations. As wind speed differs from continuous temperature variation, the hourly variation in wind speed is stepwise. Compared with temperature data of the same order of magnitude, wind speed series characteristics are more difficult to learn. When the wind speed data at Kunlun Station dropped to zero in the last 200 h, only one model, SAE LSTM, gave the closest prediction to the actual value. In the previous 200 h of the test set, the actual wind speed at Kunlun Station dropped to zero. Except for SAE LSTM, the errors shown by the remaining models exceeded 0.5 m/s. In the single-step wind speed and temperature prediction, SAE LSTM showed good stability.

To better quantify the error statistics of each model. Table 4 and Table 5, respectively, compare the five statistical indicators of the two sets of experiments in the single-step test. The bold yellow shows the best results.

It can be seen from the results in Table 4 that the NSE value of Bi LSTM is at the lowest level in each experiment. This also reflects that the model or model parameters are not suitable for forecasting such time series for the performance of the BP neural network’s temperature prediction results at Kunlun Station and Panda100 Station. The coefficient of determination is close to or even beyond the SAE LSTM model. But the poor performance on other sites shows that the algorithm requires manual tuning.

Table 5 further demonstrate that SAE LSTM shows better adaptability than other models when facing wind speed data with fewer features. In Table 5, the SAE LSTM algorithm’s performance is the best for each site and each parameter comparison. Furthermore, in the forecast of wind speed at Kunlun Station, NSE has a negative value. It shows that the BPNN model at this time is not suitable for the prediction of the sequence. Secondly, it can be seen from the statistical indicators that LSTM has the best prediction effect among the four models except for SAE LSTM. It can be seen that the advantages of long and short-term memory are—and the prediction effect of the network has been—improved after combining with SAE.

### 4.3. Experiment Two—Multi-Parameter Predictions for the Next Eight Hours

**Figure 11:**
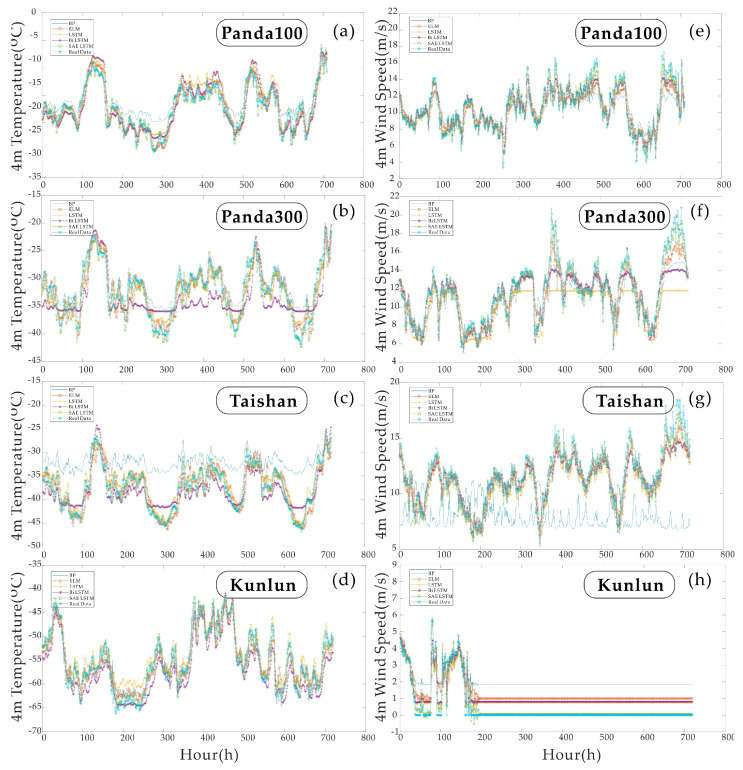
Results of multi-step predictions: (**a**–**d**) are the results of the 4 m temperature multi-step predictions for the four sites. (**e**–**h**) are the results of the multi-step prediction of the 4 m wind speed for the four areas.

Figure 11 compares the predicted value and the real value with a prediction step of 8 h. As the prediction step size increases, the correlation between data decreases, but the structure of LSTM allows more feature information to be retained. Although the rise in compensation makes it more challenging to collect wind speed data characteristics, it is observed in Figure 11. The relative trend changes are still consistent. There is no over-fitting or under-fitting phenomenon. Furthermore, the problem reflected in Figure 11 is the same as Figure 10, especially in the multi-step wind speed prediction process, except for the Panda300 station, SAE LSTM and LSTM are the two best performings. LSTM appears underfitting at panda300.

Table 6 and Table 7 list the error statistics of the wind speed and temperature prediction at the four stations with a step length of 8 h. The best results in these tables are shown in bold yellow text. Similar to the problems in the previous experiment, in the process of predicting the wind speed of Kunlun Station. In the error statistics of BPNN, NSE less than zero indicates that there is also a problem of model mismatch. However, when the single-step and multi-step prediction accuracy are compared only in terms of the coefficient of determination, the multiple temperature prediction accuracies of Kunlun Station is higher.

In comparison, the single-step prediction accuracy of the remaining three stations is more increased. BPNN predicts better than LSTM in experiments with variable step size, and the factor that causes its instability may be unadjusted parameters. However, the performance of SAE LSTM at each site is the best, regardless of the evaluation index.

The author also needs to add that in the relevant data set involving wind speed in the experiment, the wind speed data from Kunlun station have almost 20 days of 0 wind speed data (the wind speed sensor critical point wind speed is 0.2 m/s). These data are the actual standard data. Due to the low annual average wind speeds in the area, the maximum wind speed does not exceed a force three wind. It is permanently calm. This part of the data may cause the reader to be suspicious, but Dome A being the highest point in Antarctica (which is where Kunlun Station is located), the area is in a state of calm winds for up to 2 months.

The SAE LSTM combination model was found to have the best predictive performance through model presentation and data experiments. We can also try to understand it on a physical level. Variations in wind and temperature are based on the modelling theory of atmospheric physics. They are caused by local time accumulation and changes in the physical state of the spatial environment. In the case of temperature, for example, the rate of change of internal and kinetic energy with time in a matter volume element is equal to the sum of the heating rate of the external source and the power done to that matter volume element by the external source, according to the meaning expressed in the heat flow equation. As shown in Equation (18):

(18) $$c_{v}\frac{dT}{dt}=-p\frac{da}{dt}+Q+\varepsilon$$

In the formula, *T* and *p* are temperatures and air pressure, respectively; *Q* is the heating rate per unit mass of air by the external source; ***t*** is the time; ***c_v_*** is the specific constant volume heat capacity of dry air, and the value is 717 joules per kilo Kelvin (J·K^−1^·kg^−1^); *a* is the particular volume; ***ε*** is the Stokes dissipation function, which represents the dissipation rate of molecular viscosity to kinetic energy. In Equation (18), the air pressure is related to the wind speed, and the main influencing factors are the rate of change of the specific volume with time and the healing power of the external aid. Concentrated on the difference in the temperature value is expressed as the integral of the particular heat in time. For the non-adiabatic effects in the detailed section, certain factors significantly affect the temperature due to Spatio-temporal differences. As shown in both aspects, the LSTM neural network’s strength in processing time series allows for obtaining time-integrated features and trends; the SAE neural network extracts components that contain mainly non-adiabatic effects uncountable residual spaces, physical states. The combination of SAE and LSTM allows the algorithm to show good predictive performance when dealing with time series with time-varying and time-accumulative features.

## 5. Discussion

Climate predictions for East Antarctica can provide strong support for scientific research activities. For this study, it is the first time that machine learning methods have been applied to an actual meteorological survey scheme for an inland Antarctic convoy route. The four groups of experiments were introduced to reflect the new algorithm’s stability and good prediction performance from the results of five statistical indicators. Based on proposing the new algorithm, we have the following discussion:

1. In the experiment, except that the algorithm’s time complexity and space complexity is not considered much. This makes the timeliness of the conclusion to be verified. Therefore, this part will discuss in detail based on the efficiency of the five algorithms.
2. In comparing the real value and the predicted value, further data visualisation is needed. That is, whether the one-to-one correspondence between the actual value and the expected value is close to the same in the linear fitting process. In other words, whether there is over-fitting or under-fitting when the error statistics are deficient. This part will supplementary experiments based on the single-step wind speed prediction and temperature prediction of Experiment 1.
3. For multiple inputs, we choose an input combination with a Pearson coefficient close to 1. Whether to introduce other inputs will improve the accuracy of the prediction. Of course, the corresponding running time will be increased. At this time, efficiency must be considered. Therefore, we will not discuss the problem of input combinations too much here. The input combinations in this study are all combination sequences with Pearson graph correlation coefficient greater than 0.9. This ensures that the features are consistent.
4. The dataset in the experiments spanned four months. The choice of input dataset period was based on Antarctic field operation requirements. Suitable dataset lengths were selected to meet the needs of the explorers and expedition personnel for weather forecasting. To further analyse the model’s generalisability, annual data were chosen as model input data later in the long-term training process. The later stage of the model’s input data consisted of 4 m wind speed and 4 m temperature from the Kunlun station for the whole year 2020. The addition of annual data explains the zero wind speed data from Kunlun Station and further validates this new model’s applicability.

**Figure 12:**
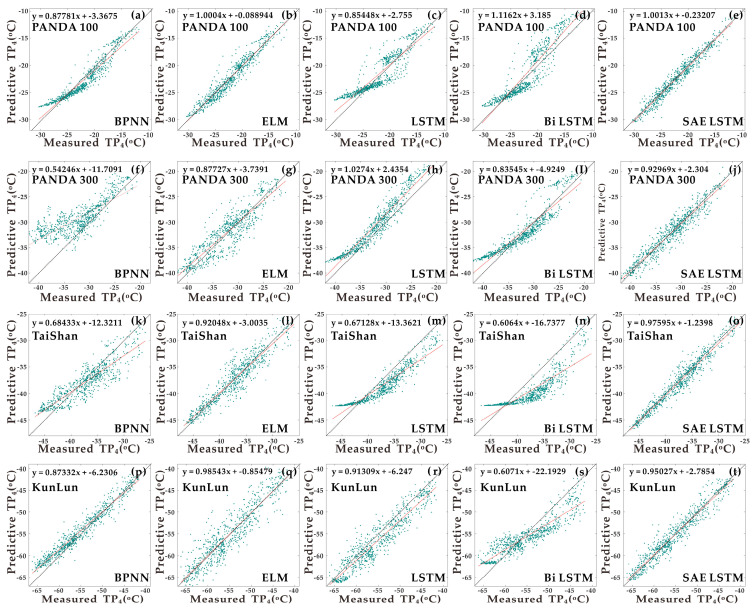
The results of a linear fit of the single-step predicted and actual values of temperature for the four stations of the five models: (**a**–**e**) represent the linear fit for the PANDA100 station; (**f**–**j**) represent the linear fit for the PANDA300 station; (**k**–**o**) represents the linear fit for the TaiShan station; (**p**–**t**) represent the linear fit for the Kunlun station.

Figure 12 is a statistical test of model prediction results. The coefficient of measurement (R-squared) and the slope of the proper function of the prediction scatter plot are commonly used parameters to judge the prediction results. The closer these two parameters are to 1, the better the prediction result. Based on this observation, Figure 12a–t can be obtained.

According to the obtained Figure 12, it can be seen that in the fitting process of each site, the slope of the right line of the ELM neural network in most cases is closer to 1, followed by SAE LSTM. But the stability of SAE LSTM is better, in the single-step temperature prediction of each site. The slopes of the fitted straight lines are all greater than 0.92. In addition to this, combined with the correlation coefficient R^2^, it can be seen that the prediction results of SAE LSTM are more stable, while ELM will fluctuate. The product is very biased. The other three models only performed well in one or two experiments. Therefore, Figure 12 reflects the actual situation of the forecast more clearly. It further illustrates that after SAE combined with LSTM, the LSTM network is more stable, and the prediction accuracy is higher.

**Figure 13:**
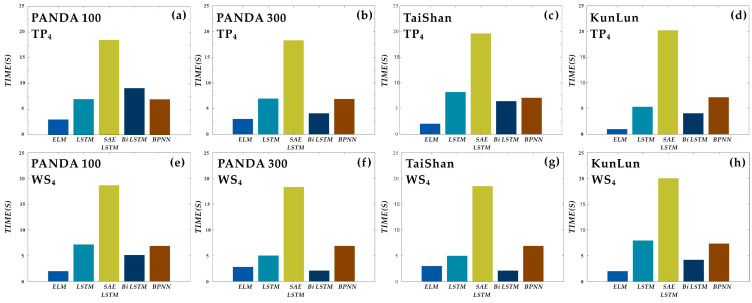
Single-step prediction experiment 4 m temperature versus wind speed at four sites: (**a**–**d**) are comparisons of individual model times for single-step prediction of 4 m temperature at four locations; (**e**–**h**) are comparisons of personal model times for single-step prediction of 4 m wind speed at four sites.

Figure 13 shows a comparison of the training times of the models in the experimental panda100 site 4 m temperature prediction experiment. It can be seen from the figure that the size of the data sample affects the running time of each model. For the analysis of algorithmic complexity, the complexity of the forward/backward propagation of the neural network is proportional to the number of parameters when looking at the complexity of a single iteration, i.e., O(N) when using the Big O analysis, where N is the size of the input data. The primary analysis method used in this experiment is to evaluate the code runtime environment and the corresponding hardware, combined with the actual runtime of each model algorithm as an evaluation metric.

When using the SAE LSTM model, the running time is longer than with other models. However, the prediction accuracy of LSTM has improved significantly. This increase in the prediction accuracy of the algorithm inevitably leads to an increase in complexity and training time. However, run times of less than 30 min can be used for action planning in the field. However, considering that ELM consumes the shortest time per training and test of any model and that the slope of the algorithm’s fit is closest to 1 in the linear fitting process, there are some cases where it exhibits large deviations from the norm, the ELM neural network can be considered in cases where the shortest time is considered.

Finally, the authors present wind speed and direction data from Kunlun Station for the full year 2020, supplemented by a wind speed prediction experiment with the full-year data as input.

**Figure 14:**
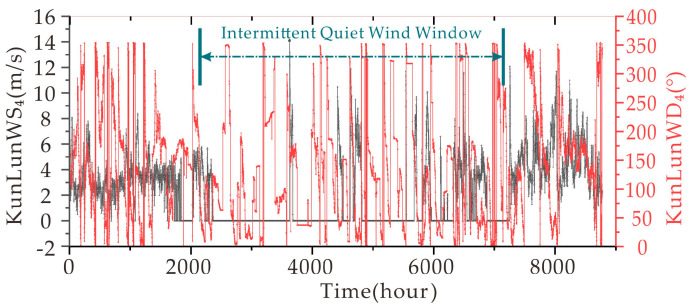
The 4 m wind speed and direction at Kunlun weather station for the whole year of 2020.

The first consideration concerning the appearance of static wind data is the sensor’s operating status, which can be determined in two ways. The first is to observe whether the angle of the wind sensor changes on a quiet wind day, as a quiet wind is not the absence of wind, but there will be vertical winds as well as winds of less than 0.3 m/s. In Figure 14, the wind speed and direction data for the whole year at Kunlun station are illustrated, where the period indicated by the blue illustrated double arrow is the intermittent quiet wind window. At the end of the year 2020 data near the 7000-h position on the axis, the windy season was entered, and the sensor returned wind speed values. Combining these two points would suggest that the zero wind speed input data from the earlier experiments were not the result of a damaged sensor.

**Figure 15:**
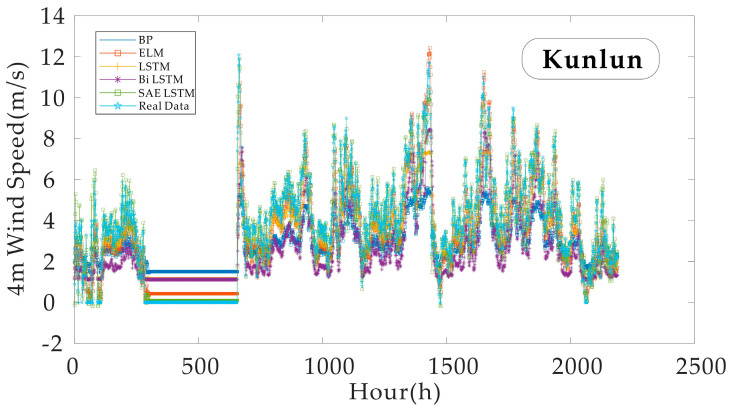
A comparison of the models’ predictions in the Kunlun Station 4 m wind speed data throughout the year as input data test set.

**Figure 16:**
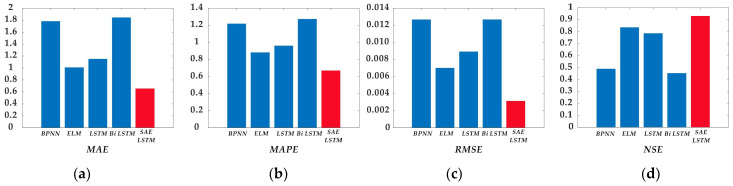
Comparison of statistical errors in prediction results for 4 m wind speed data at Kunlun Station throughout the year. (**a**) Five model statistical errors under the MAE criterion; (**b**) Five model statistical errors under the MAPE criterion; (**c**) Five model statistical errors under the RMSE criterion; (**d**) Five model statistical errors under the NSE criterion.

A span of 8763 h was entered into the data set during the later full-year data analysis. The start and end dates are 1 January 2020, and 31 December 2020. Figure 15 shows a comparison between the test set and the input data. It can be seen that the predicted values of WS4, the subject of the SAE LSTM model, follow the trend of the real values better. To illustrate the problem even further, the authors compare four error statistics in Figure 16. Under each statistical criterion, the new model reflects advantages consistent with the findings of previous experiments. This complementary experiment reaffirms the strength of the model for dealing with the prediction of surface weather under multi-sensor data input conditions in the region.

## 6. Conclusions

This study aims to create a new neural network model to be applied to future short-term parameter predictions at Antarctic multi-sensor weather stations to achieve accurate and efficient camp weather predictions. This will ensure the safety of inland convoys when camping and operating along the route. From these experiments and discussions, the following conclusions were drawn.

(1) In the algorithm training experiments, when selecting the input variables for the weather station, variables with a correlation higher than 0.9 can be chosen as input variables according to the Pearson diagram.
(2) When performing ambient weather prediction in the field, it is concluded that the SAE LSTM neural network model is superior to the other four neural network models in terms of decision coefficient and slope of fit. The network solved the gradient explosion problem and obtained more data series characteristics with SAE. However, the model with the shortest time and better fit was the ELM neural network model.
(3) There was little variability in the model’s performance at each site during the multi-group experiments. Therefore, the model is feasible for meteorological prediction and environmental assessment of the East Antarctic Inland Line fleet.

The Antarctic interior is the primary location for scientific observation activities and scientific experiments in Antarctica. In addition to the harsh environment, which creates excellent conditions for the experimental requirements, the uncertain climate adds new difficulties to the experiments. This is why the prediction of near-Earth weather in a small area is a must for the field staff to ensure that the investigations are carried out. In the past, however, erroneous forecasts had often led to inevitable losses during periods when ground-based sensor networks and machine learning methods were lacking. The deep learning neural network model based on a novel weather station proposed in this paper provides a new prediction tool for inland camp weather forecasting. Compared to the commonly used BPNN and LSTM neural networks, the new algorithm solves the gradient explosion problem and extracts more sequence features via the SAE network.

If scenarios or data types are changed, data cleansing and parameterisation are done if necessary. Artificial intelligence brings shortcuts and new methods to research in related areas. In future research, a camp-wide digital twin network in the Antarctic could be considered. Equipment such as LORA could be regarded as to make an extensive, fine-grained observation of the operational area. Along with increasing the density of the sensor network layout, the computing power of the corresponding core hardware and the optimisation of the algorithms must also be completed. In future studies, it is expected that sensor networks will be set up at permanent Antarctic stations such as Zhongshan, Kunlun or Taishan, where the digital twin will be used to provide the crew with real-time information on the future operational status of the camps and equipment observations.

## Figures and Tables

**Table 1:** Details of selected stations.

Station ID	Latitude	Longitude	Altitude	Period	Data Volume
Panda 100	70°13′02.26″ S	76°39′14.70″ E	1352 m	1 January 2020–30 April 2020	2893
Panda 300	71°59.510″ S	77°56.460″ E	2344 m	1 January 2020–30 April 2020	2893
Tai Shan Station	73°51′48.7″ S	76°58′43.5″ E	2626 m	1 January 2020–30 April 2020	2904
Kun Lun Station	80°25′41.7″ S	77°07′27.75″ E	4093 m	1 January 2020–30 April 2020	2904

**Table 2:** Computer configuration.

Name	Detailed Settings
Hardware	Desktop computer
CPU & Frequency	Intel(R) Core (TM)I5 10,600 KF 4.10 GHz
RAM	DDR4 16 G 2666 MHz
Hard drive	4 T
Software & Operating system	MATLAB R2019a, Windows 10

**Table 3:** Parameter setting of the neural networks.

Algorithm	Parameter Settings
BP	MEp = 250, GT = 1.0 × 10^−7^, H1 = 10, H2 = 10. AF = S
ELM	ME = 1000, AF = S
LSTM	MEp = 250, GTd = 1, ILR = 0.01, LRDP = 125, LRDF = 0.1, V = 0, L2R = 0.0001
Bi LSTM	MEp = 250, GTd = 1, ILR = 0.01, LRDP = 125, LRDF = 0.1, V = 0, L2R = 0.0001
SAE-LSTM	MEp = 250, GTd = 1, ILR = 0.01, LRDP = 125, LRDF = 0.1, V = 0, ETF = satlin, DTF = purelin, L2R = 0.0001, L2WR = 4, SP = 0.6

**Table 4:** One-hour temperature performance results from station Panda 100 to Kun Lun station.

Station ID	Algorithm	*MAE*	*MAPE*	*RMSE*	*R* ^2^	*NSE*
Panda 100	BPNN	1.068426	0.0059516	1.383505	0.90225	0.872779
	ELM	0.910392	0.0044656	1.074031	0.9294	0.923329
	LSTM	1.174858	0.0059241	1.737256	0.81599	0.799403
	Bi LSTM	1.270804	0.0057111	2.011734	0.84367	0.731009
	SAE LSTM	0.867028	0.0040089	0.955108	0.94698	0.939368
Panda 300	BPNN	1.78986	0.011883	4.15952	0.64917	0.223129
	ELM	1.29860	0.003540	2.09222	0.81088	0.803449
	LSTM	1.32287	0.008062	2.06952	0.92689	0.807689
	Bi LSTM	1.24206	0.004531	1.87724	0.84813	0.841764
	SAE LSTM	1.03392	0.000564	1.36253	0.91689	0.916640
Tai Shan Station	BPNN	1.34427	0.00349	2.24386	0.79107	0.764348
	ELM	1.11192	0.00077	1.63781	0.87668	0.874453
	LSTM	1.37566	0.00567	2.19589	0.87488	0.774316
	Bi LSTM	1.67569	0.00809	3.22362	0.72215	0.513632
	SAE LSTM	0.93141	0.00346	1.15443	0.94412	0.937624
Kun Lun Station	BPNN	1.14188	0.004533	1.681002	0.94461	0.924677
	ELM	1.42503	0.001038	2.612523	0.84242	0.818068
	LSTM	1.47131	0.005919	2.596735	0.87888	0.820260
	Bi LSTM	1.57854	0.002777	3.051582	0.81268	0.751779
	SAE LSTM	1.10634	0.000626	1.612757	0.93113	0.930669

**Table 5:** One-hour wind speed performance results from station Panda 100 to Kun Lun station.

Station ID	Algorithm	*MAE*	*MAPE*	*RMSE*	*R* ^2^	*NSE*
Panda 100	BPNN	1.22455	0.011725	1.95765	0.8678	0.47831
	ELM	1.05181	0.008427	1.41745	0.80165	0.72650
	LSTM	0.82797	0.003602	0.89648	0.89947	0.89060
	Bi LSTM	0.82225	0.003769	0.88920	0.90431	0.89237
	SAE LSTM	0.78907	0.003110	0.84079	0.90442	0.90376
Panda 300	BPNN	1.24344	0.009894	1.97035	0.68139	0.62930
	ELM	0.89355	0.004935	1.02083	0.90256	0.90050
	LSTM	0.85351	0.009037	1.00813	0.9475	0.90296
	Bi LSTM	1.05321	0.011261	1.75309	0.82914	0.70654
	SAE LSTM	0.73761	0.003859	0.72451	0.95092	0.94988
Tai Shan Station	BPNN	1.08757	0.010332	1.58745	0.81705	0.590697
	ELM	1.04612	0.007937	1.45715	0.68456	0.655129
	LSTM	0.797037	0.001467	0.81375	0.8935	0.892445
	Bi LSTM	1.190315	0.011844	1.92852	0.91214	0.395920
	SAE LSTM	0.765848	0.003389	0.75822	0.91707	0.906625
Kun Lun Station	BPNN	1.49075	0.009587	2.34532	0.74122	−2.448817
	ELM	0.972161	0.011266	1.00716	0.84713	0.363992
	LSTM	0.967613	0.007343	0.982736	0.89832	0.394464
	Bi LSTM	0.795267	0.010005	0.683786	0.91114	0.706839
	SAE LSTM	0.374660	0.006919	0.381148	0.91215	0.908914

**Table 6:** Eight-hour temperature performance results from station Panda 100 to Kun Lun station.

Station ID	Algorithm	*MAE*	*MAPE*	*RMSE*	*R* ^2^	*NSE*
Panda 100	BPNN	1.240293	0.009947	2.0031519	0.95165	0.73389830
	ELM	1.020948	0.001737	1.3268095	0.89174	0.88325535
	LSTM	1.241437	0.005180	1.8603498	0.83125	0.77048605
	Bi LSTM	1.170576	0.002586	1.7146309	0.85193	0.80503297
	SAE LSTM	0.799223	0.002637	0.8234433	0.95677	0.95503369
Panda 300	BPNN	1.31158	0.007194	2.228846	0.89663	0.777248
	ELM	1.27735	0.001690	2.042462	0.81483	0.812945
	LSTM	1.17435	0.002504	1.719967	0.88167	0.867352
	Bi LSTM	1.74283	0.006739	3.508384	0.53774	0.448080
	SAE LSTM	0.99339	0.005022	1.279989	0.9446	0.926536
Tai Shan Station	BPNN	2.34451	0.01455	6.29224	0.78792	−0.848494
	ELM	1.31529	0.00222	2.17819	0.78758	0.778487
	LSTM	1.13080	0.00570	1.55644	0.92385	0.886897
	Bi LSTM	1.42752	0.00432	2.30887	0.77723	0.751110
	SAE LSTM	0.89957	0.00208	1.08710	0.94596	0.944825
Kun Lun Station	BPNN	1.22528	0.003049	1.878726	0.92473	0.906087
	ELM	1.35242	0.001992	2.245464	0.87836	0.865843
	LSTM	1.34439	0.006172	2.277129	0.92849	0.862033
	Bi LSTM	1.58325	0.006873	3.026850	0.86167	0.756228
	SAE LSTM	1.10337	0.002384	1.608872	0.93119	0.931128

**Table 7:** Eight-hour wind speed performance results from station Panda 100 to Kun Lun station.

Station ID	Algorithm	*MAE*	*MAPE*	*RMSE*	*R* ^2^	*NSE*
Panda 100	BPNN	1.08436	0.009406	1.54300	0.86431	0.67647
	ELM	1.05223	0.008240	1.41236	0.78459	0.72894
	LSTM	0.83556	0.003118	0.90966	0.89392	0.88756
	Bi LSTM	0.83757	0.005422	0.93312	0.89297	0.88168
	SAE LSTM	0.78530	0.004809	0.83388	0.90745	0.90551
Panda 300	BPNN	1.17645	0.009566	1.81272	0.72871	0.68662
	ELM	1.02109	0.007907	1.36177	0.8481	0.82314
	LSTM	1.35739	0.014995	2.57778	0.62156	0.36627
	Bi LSTM	0.99511	0.009417	1.58452	0.80047	0.76055
	SAE LSTM	0.75632	0.005108	0.76305	0.94759	0.94447
Tai Shan Station	BPNN	2.04921	0.021993	4.87583	0.48208	−2.868091
	ELM	0.92340	0.007182	1.10104	0.82362	0.80277
	LSTM	0.97317	0.010454	1.17773	0.90376	0.77432
	Bi LSTM	0.87164	0.007578	1.02533	0.88236	0.82896
	SAE LSTM	0.77561	0.005032	0.77643	0.90655	0.90192
Kun Lun Station	BPNN	1.27826	0.007470	1.71073	0.88838	−0.88725
	ELM	0.99206	0.011990	1.04184	0.82365	0.30005
	LSTM	0.84939	0.012332	0.77218	0.8901	0.61550
	Bi LSTM	0.87456	0.010047	0.81500	0.89932	0.57166
	SAE LSTM	0.41495	0.006716	0.37476	0.91362	0.90943

## Acronyms

TP_4_	Temperature at 4 m altitude
TP_2_	Temperature at 2 m altitude
TPL_4_	Temperature Lowest at 4 metres altitude
TPL_2_	Temperature Lowest at 2 metres altitude
HM_4_	Humidity at 4 m altitude
HM_2_	Humidity at 2 m altitude
WS_4_	Wind Speed at 4 m altitude
WS_2_	Wind Speed at 2 m altitude
WD_4_	Wind Direction at 4 m altitude
WD_2_	Wind Direction at 2 m altitude
WSM_4_	Wind Speed Maximum at 4 m altitude
WSM_2_	Wind Speed Maximum at 2 m altitude
BT_V_	Battery Voltage
CELL_T_	Controller Cell Temperature
BPNN	Back Propagation Neural Networks
ELM	Extreme Learning Machine
LSTM	Long Short-Term Memory
AE	Auto Encoder
SAE	Stacked Auto Encoder
SAE LSTM	Stacked Auto Encoder Long Short-Term Memory
Bi LSTM	Bi-directional Long Short-Term Memory
